# Developing model biobanking consent language: what matters to prospective participants?

**DOI:** 10.1186/s12874-020-01001-2

**Published:** 2020-05-15

**Authors:** Laura M. Beskow, Catherine M. Hammack-Aviran, Kathleen M. Brelsford

**Affiliations:** Center for Biomedical Ethics and Society, Vanderbilt University Medical Center, 2525 West End Avenue, Suite 400, Nashville, TN 37203 USA

**Keywords:** Informed consent, Research ethics, Patient/participant perspectives, Biobanking, Precision medicine research

## Abstract

**Background:**

Efforts to improve informed consent have led to calls for providing information a reasonable person would want to have, in a way that facilitates understanding of the reasons why one might or might not want to participate. At the same time, advances in large-scale genomic research have expanded both the opportunities and the risks for participants, families, and communities. To advance the use of effective consent materials that reflect this landscape, we used empirical data to develop model consent language, as well as brief questions to assist people in thinking about their own values relative to participation.

**Methods:**

We conducted in-person interviews to gather preliminary input on these materials from a diverse sample (*n* = 32) of the general population in Nashville, Tennessee. We asked them to highlight information they found especially reassuring or concerning, their hypothetical willingness to participate, and their opinions about the values questions.

**Results:**

Consent information most often highlighted as reassuring included the purpose of the biobank, the existence and composition of a multidisciplinary oversight committee, the importance of participants’ privacy and efforts to protect it, and controlled access to a scientific database. Information most often highlighted as concerning included the deposition of data in a publicly accessible database, the risk of unintended access to data, the potential for non-research use of data, and use of medical record information in general. Seventy-five percent of participants indicated initial willingness to participate in the hypothetical biobank; this decreased to 66% as participants more closely considered the information over the course of the interview. A large majority rated the values questions as helpful.

**Conclusions:**

These results are consistent with other research on public perspectives on biobanking and genomic cohort studies, suggesting that our model language effectively captures commonly expressed reasons for and against participation. Our study enriches this literature by connecting specific consent form disclosures with qualitative data regarding what participants found especially reassuring or concerning and why. Interventions that facilitate individuals’ closer engagement with consent information may result in participation decisions more closely aligned with their values.

## Background

Recent changes to U.S. federal regulations governing the conduct of much human-subjects research focused extensively on informed consent. With the goal of enabling better-informed decisions about participating in research [1] — including biospecimen research in particular [2] — new rules require that prospective participants be provided with information that a reasonable person would want to have, and that it be presented in sufficient detail and organized in a way that facilitates understanding of the research that will be conducted and the reasons why one might or might not want to participate.

At the same time, rapid advances in large-scale genomic research, including the ability to collect and analyze multidimensional genomic, medical, lifestyle, and behavioral data, have expanded both the opportunities and the risks for participants, families, and communities [3, 4]. These lead to value-laden issues surrounding research participation, i.e.*,* factors that in some cases cannot be quantified but rather must be considered by individuals relative to their own personal circumstances [5].

To advance the use of effective consent materials that meet new regulatory requirements and reflect the research landscape, we developed model consent language based on the results of our long-standing programs of research on biobanking consent and the protection of participants in precision medicine research. Further, we constructed a brief set of questions intended to assist people in thinking about their own values relative to biobank participation. We gathered preliminary input on these materials from a diverse sample of the general population in Nashville, Tennessee, including which information they found especially reassuring or concerning, their hypothetical willingness to participate in the biobank as described, and their opinions about the helpfulness of the values questions.

## Methods

### Materials development

We developed our model consent language, which refers to a hypothetical “Million American Study,” based on best practice guidelines for biobanking [6–8]; information required by recently revised federal regulations (45 CFR 46.116); and the results of our own series of studies to simplify biobanking consent and improve comprehension [9–14], which over time involved more than 2100 members of the general public and nearly 80 professionals with biobank-related expertise in cognitive interviews, in-depth interviews, a randomized survey, and a formal Delphi process. Notably, we also incorporated key findings from in-depth interviews we conducted with a diverse group of 60 thought leaders on the benefits, risks, harms, and protections in precision medicine research [5, 15, 16], as well as extensive analysis of federal and state protections available for precision medicine research [17]. In constructing our language, we followed principles of plain-language writing, such as using common, lay words; writing in first person, active voice; limiting sentence length; addressing only one main idea per paragraph; using clear organization and formatting with descriptive headings; and ensuring adequate white space [18]. Further, although consent forms often describe risks and protections separately, we combined these with the goal of aiding prospective participants in grasping the extent to which risks are mitigated and where potential gaps remain. Prior to gathering the input reported here, our consent form was reviewed by all members of our multidisciplinary research team, as well as two colleagues with substantial expertise in human subjects protections, and cognitively tested with two lay individuals. See Additional file 1: **Appendix S1** for a complete copy of our consent form with readability characteristics.

In addition, our thought leader interviewees typically described the risks of participation as inherently hard to quantify and value-laden—often remarking that “It depends on the particular participant’s situation” or “It really depends on the person” [5]. Accordingly, we drew upon the results of these interviews as well as decision aid literature [19, 20] to devise a brief set of questions (Table 1) to assist individuals in evaluating consent information in light of their own circumstances.

**Table 1:** Values questions (as presented to interview participants) The table below summarizes some of the things you learned from the consent form. To help decide whether you want to participate in the Million American Study, think about each statement and mark how much it matters to you. Don’t worry about anyone seeing your answers—this is just to help you think through for yourself what you might like to do

If I participate in this project. . .	How much does this matter to you?				
	Not at all			Very much	
There is a chance that someone without permission could get access to my private information.	1	2	3	4	5
My data may reveal things about my family.	1	2	3	4	5
My data (along with others’) may help researchers learn things that could improve health care for people in the future.	1	2	3	4	5
There are laws to protect me, but they have gaps.	1	2	3	4	5
I probably will not learn anything helpful to my own health or medical care.	1	2	3	4	5
The risks could change over time.	1	2	3	4	5
My samples and information could be used for research I do not like or would rather not support.	1	2	3	4	5

### Feedback from prospective participants

To elicit input on the model form and values questions, we conducted in-person qualitative interviews among members of the general public in Nashville, Tennessee.

#### Participants

We hired a professional research firm to assist with recruitment. English-speaking adults at least 21 years of age were eligible. Those who had participated in more than two medical research studies in the past year, or whose jobs involved health-related research or the provision of healthcare, were excluded. Among eligible participants, we used purposive selection to maximize demographic diversity. Most interviews took 45–60 min to complete and participants were paid $75 for their time.

#### Data collection

Two research team members conducted the interviews in October 2018. Participants reviewed a study information sheet, provided verbal agreement to participate and for audio recording, and were then asked to read the model consent form. After this initial review (**Time 1**), we asked participants to indicate on a 6-point scale how likely they would be to participate in the hypothetical Million American Study.

Next, we asked participants to highlight sentences in the consent form they found especially reassuring or concerning. Specifically, half of participants, broadly stratified by demographic characteristics, were first instructed, “Use this marker to highlight any parts of the form that you especially like or find especially reassuring.” After they finished, we asked them to tell us more about each sentence highlighted. We then gave them a clean copy of the consent form and repeated the process, this time asking them to highlight any parts of the form they especially disliked or found especially concerning. The other half of participants completed the same set of activities except that we asked them to highlight concerning sentences first, followed by reassuring sentences, to control for any ordering effects (e.g.*,* fatigue or inattention to the task over time). After completing both highlighting activities (**Time 2**), we again asked participants to rate their willingness to participate.

Finally, we asked participants to provide feedback on the values questions. Rather than collecting their answers to the questions per se, our instruction was, “We’re wondering if thinking through questions like these might be helpful for people deciding whether or not they want to participate in a biobank in real life. Go ahead and fill these out, but this is only for you—you don’t have to show me your answers. The point is only to help people think these things through for themselves.” We asked their opinions about whether these types of questions in general would be helpful for people contemplating biobank participation, as well as their input on our specific questions.

To complete the interview (**Time 3**), we asked participants to rate their hypothetical willingness to participate one last time.

#### Data analysis

Highlighted sentences, ratings of willingness to participate at the three timepoints, and categorical reactions to the values questions (helpful, neutral/mixed, not helpful) were transferred to Microsoft Excel (2016). Data entry was checked for accuracy by multiple team members and basic descriptive analyses were performed in Excel. Below we describe the sentences in the consent form highlighted most frequently; see Additional file 1: **Appendix S2** for data concerning every sentence in the form (as well as justification for the content relative to regulatory requirements).

Audio recordings were professionally transcribed. We used NVivo 12 (QSR International) and a standard iterative process [21]—including two independent coders who reached ≥80% inter-coder agreement—to code and analyze the data. Narrative segments presented here (along with participant IDs) are exemplary of frequently mentioned ideas unless otherwise noted.

## Results

### Participant characteristics

Overall, our participants (*n* = 32) were diverse in terms of age, gender, race/ethnicity, education, income, and healthcare visits (Table 2).

**Table 2:** Participant characteristics (*n* = 32)

	n	(%)
Age Group (years)		
< 25	1	(3)
25–34	5	(16)
35–44	7	(22)
45–54	11	(34)
55–64	4	(13)
65 +	4	(13)
Gender		
Male	15	(47)
Female	17	(53)
Race		
Black or African American	9	(28)
White	23	(72)
Ethnicity		
Hispanic, Latinx, or of Spanish origin	5	(16)
Education		
High school graduate	6	(19)
Associate’s degree	11	(34)
Bachelor’s degree or higher	15	(47)
Household Income		
< $25 k	3	(9)
$25–49.9 k	11	(34)
$50–74.9 k	6	(19)
$75–99.9 k	3	(9)
$100–124.9 k	5	(16)
$125–149.9 k	3	(9)
$150 k +	1	(3)
Healthcare Visits (in past year)		
< 5	10	(31)
5–9	11	(34)
10 +	11	(34)

### Sentences highlighted as reassuring

Of the 215 sentences in the consent form, participants on average highlighted 28 as especially reassuring (range: 0–98) (Additional file 1: **Appendix S3**). Sections of the form containing sentences most commonly identified as reassuring (by ≥20% of participants) included (Table 3):

**Table 3:** Sentences commonly highlighted as reassuring^a^

Section / Subsection^*^	Sentences Highlighted
**WHY IS THIS PROJECT BEING DONE?**	
Researchers will use the stored materials in future studies on health and disease. Through such studies, they hope to find new ways to detect, treat, and maybe prevent or cure health problems.	
**WHO IS DOING THIS PROJECT?**	
There is a Steering Committee to watch over the project and make sure we are doing things the right way. This includes researchers, doctors, lawyers, ethics experts, and government officials. It also includes patients and members of the public to help speak for people who take part.	
**WHAT WILL YOU ASK ME TO DO?**	
We will get a blood sample from you.	
We will use a needle to draw about 3 tablespoons of blood from your arm.	
**WHAT WILL YOU DO WITH MY SAMPLE AND INFORMATION?**	
We will store all the samples and information in a central place.^*^	
We will store your sample and information, along with those from all the other people who take part, at a secure location in Norfolk, Virginia. There will be many layers and kinds of safeguards to help keep the materials safe.	
We will not give researchers anything that directly identifies you.^*^	
When we store the materials, we will remove names and other identifiers. We will replace them with code numbers.	
Only a few project staff can see the list, and they sign a pledge to keep it secret.	
People who study the samples and information will not know who you are. We will give them materials labeled with only the code numbers.	
Researchers will do many kinds of studies to learn about health and disease.^*^	
• What causes people to be healthy or have a disease?	
• Why do diseases and treatments affect people differently?	
• How do basic biology, lifestyle, and environment work together to affect health?	
Some of the studies may lead to new products, such as drugs or tests for diseases.	
Researchers will have two ways to access the stored materials.	
We will put some information in a **public database** that anyone can look at. This database will not contain names or other direct identifiers. Further, it will not allow anyone to see information about just one person. It will only show information at a group level (for instance, for the group of people in the Million American Study who have heart disease).	
We will also make a **scientific database**. Like the public one, this database will not contain names or other direct identifiers.	
Access to the scientific database will be strictly controlled. Researchers who want to use it must first complete ethics training. Then they must apply to do their specific study. An Access Committee will review each request. If a study is approved, the researcher must sign a pledge to use the materials only for that study. They also promise to keep the materials secure and not try to figure out who you are.	
**ARE THERE ANY BENEFITS?**	
The main reason you may want to join is to help researchers learn things that could improve health care for people in the future.	
**WHAT ARE THE RISKS AND HOW WILL I BE PROTECTED?**	
Someone could identify you.	
Your privacy is very important to us and we will make every effort to protect it. We will keep everything in a secure place and label it only with a code. We will not give out anything that tells who you are. Nobody will know just from looking at the databases that the information belongs to you.	
We will follow federal rules designed to make sure only the right people see your data. These include limiting and tracking who has access, as well as passwords, encryption, and other safeguards. We will tell you if someone sees the data who was not supposed to.	
The stored materials could be used for studies you do not like.	
The goal of the Million American Study is to make discoveries that improve health for everyone.	
You can learn about the studies being done on the Million American Study web site [URL].	
You have the right to leave the project at any time (see the part below, “What are my options?”).	
Your sample and information could be of interest for reasons other than research.	
Federal laws also make it illegal for most **employers** to use your genetic information against you.	
There are federal laws that require us to refuse to give out information that identifies you, even if ordered to by a court or judge, without your okay. Still, we must follow laws that require us to report certain things to **state officials**.	
The stored materials could suggest information about your family.^*^	
*[Concluding paragraphs of Risks & Protections section]*	
The federal laws we described protect you no matter where you live.	
We will tell you if we learn of anything that might change your decision to take part.	
**ARE THERE ANY COSTS OR PAYMENTS?**	
Some research may lead to new products, such as drugs or tests for diseases.	
**WILL I GET THE RESULTS OF STUDIES ON MY SAMPLE AND INFORMATION?**	
There is a small chance that researchers could find something that might be very important to your health or medical care right now. At the end of this form, you can tell us whether you want us to try to contact you if this happens.	
Getting results may affect your privacy risks.	
We will not give information to insurance companies.	
**WHAT ARE MY OPTIONS?**	
Taking part in the Million American Study is your choice. You can choose to join or not. If you decide to join, you can change your mind at any time.	

^a^Sentences highlighted as reassuring by 6 (~ 20%) or more participants; see Additional file 1: Appendix S2 for the complete consent form and highlighting counts

^*^Section / Subsection information is provided primarily as a navigational aid for readers; instances where the heading itself was frequently highlighted are denoted with an asterisk

Why is this project being done? Sentences describing the purpose and goals of the biobank were particularly reassuring:

I like the part where it says, “Research studies, they help to find new ways to detect, treat or maybe even prevent and cure health problems.” I think it gives people a sense that they’re helping the cause. (08)

Who is doing this project? The description of the biobank governance structure, including the involvement of both professional and community representatives, was also particularly reassuring:

You do feel safe giving your information, especially the inclusions of researchers and doctors. You got experts on the topic who know what they’re talking about. I like that it’s not just lawyers and ethics experts and government officials, but it’s also people that would be doing these types of research. (03)

To be honest with you, [I’m reassured] that it’s actually got patients as well as members of the public, that it’s not just the ivory tower folks. (04)

What will you ask me to do? Some participants were reassured by details about the blood draw:

It was so specific, saying three tablespoons of blood from your arm. Like, exactly how much and where they’re taking it from. A lot of people get nervous about stuff like that, and I feel like being super specific about it ... would set me up to be trusting and open to the study because they’re being so super transparent. (12)

What will you do with my samples and information? This section of the consent form included information about several topics that participants found reassuring:

#### Central storage

The description of secure storage in a central location with multiple safeguards was particularly reassuring:

It’s going to be all in one location, it’s going to be secure … There will be a lot of safeguards in place, so it’s just not going to be easily accessible. (17)

#### Restricted access to identifiers

Sentences describing that materials would be stored in coded form and that researchers would not receive direct identifiers were also particularly reassuring:

I like that. You become a number and … only a few project staff can see the list. All that is safeguarding and stuff. Just that whole section is more safety. That they don’t know whoever you are, that you’re just a number. (13)

#### Use of stored materials for research

Many participants found general descriptions of the kinds of research that would be done reassuring:

‘Why do diseases and treatments affect people differently and how do basic biology, lifestyle and environment work together to affect health.’ I think this is one of my favorite ones out of all of them because it’s showing … the breakdown of it … It’s really interesting to see—this shows me what they’re looking for. (24)

This included the possibility that new products might be developed:

I was impressed, some of the studies may lead to new products such as drugs or tests for diseases, which would be a great help. (19)

#### Public-access database

Some participants were reassured by the description of a publicly-accessible database containing no direct identifiers:

It might be interesting for some people to see what other people have. Maybe there's a bunch of people in a certain town that all of a sudden there's an upswing of cancer or things like that … A public database would make that information available to the public instead of hiding it. (32)

#### Controlled-access database

Even more commonly, participants were reassured by the description of a scientific database and the procedures required for researchers to access it:

I think that’s just another way of them trying to reassure me that they’re going to be as professional and discreet as possible and make sure the information doesn’t fall into the wrong hands. (21)

Are there any benefits? Some participants were reassured that the research could improve health care for people in the future, explaining that “we desperately need to improve healthcare” (09).

What are the risks and how will I be protected? Sentences describing basic efforts to protect privacy and confidentiality were particularly reassuring, including details about federal rules to protect privacy/confidentiality and the use of codes, passwords, encryption, and other safeguards:

Privacy almost doesn’t exist with the tools and stuff that are out there, so at least they’re making an effort … I like to see the multi-layered security. (29)

Other commonly highlighted sentences in this section included:

- *Protection against objectionable use:* Many participants were reassured by a sentence reemphasizing the objective of the biobank: “[The sentence that says] ‘The goal of the Million American Study is to make discoveries that improve health for everyone’ – to me, that makes me feel like they actually care.” (24)
- *Availability of information about studies and aggregate results:* Some were reassured that they could learn about studies being done through the biobank. As one commented, “I think it’s a great idea to be able to go to a website to pull up the studies. It’s gonna be done as a group anyway. It’s not being done as an individual.” (06)
- *Right to withdraw:* Many participants were reassured by a sentence underscoring their right to withdraw: “I liked that they laid out that you have the right to leave the project at any time, that you’re not stuck in the project if you feel like there’s been some sort of violation or if you don’t feel the need to participate anymore.” (10)
- *Genetic Information Nondiscrimination Act (GINA)*: Several were reassured that “we have laws that employers can’t use our genetic information against us.” (13)
- *Certificates of Confidentiality:* Some participants were reassured by information about Certificates. As one elaborated, “[I like] the fact that federal law keeps you from giving stuff to law enforcement or lawyers or what not. I do like the fact that you remind folks that you are still required to report … instances of things that are a threat to public health. Whether it be, ‘Oh, this guy’s got smallpox, this guy’s got whatever.’” (04)
- *Familial implications:* Some were reassured that stored materials could suggest information about their family. As one explained, “Certainly, if I were to participate in something like this and they were like, ‘Hey, we found out this guy has the potential to have cancer in his family, or to have depression … or whatever,’ it would be good to know, especially for my children. To be able to say, ‘Hey, your dad has this, so make sure y’all keep an eye on it.’” (29)
- *Federal laws as minimum protection:* Some participants were reassured that federal laws apply no matter where they live: “It’s also good they’re telling you that federal laws protect you wherever you are, and I do realize some states have their own laws for more protection but never less protection. That’s a good point, too.” (06)
- *Unknown, future risks:* Finally, some highlighted the assurance that they would be informed of anything that might change their decision to take part: “I like that it’s not just out there for you to keep up on all that, but they say that they’ll inform you if anything changes.” (03)

Are there any costs or payments? Consistent with the section on ‘Use of stored materials for research,’ some participants were reassured by the prospect of research leading to new products:

It’s something positive. Any reasonable person would want a cure to cancer or some other disease, Alzheimer’s or something like that … That’s why I think describing the goal of the database is very important and reassuring. (23)

“Research may lead to new products, such as drugs and tests for diseases,” which is great. We need more of those. (25)

Will I get the results of studies on my sample and information? Some participants were reassured by the option of being contacted in the unlikely event that researchers found something of immediate importance for their health:

I’d want to know. Hey, if you found out I’ve got something, don’t keep it to yourself … Let me know that there’s something I might want to go to my physician and have checked out. (32)

Many were reassured to learn that such information would not be given to insurance companies: “It’s kind of a hot topic right now of being denied for pre-existing or known things.” (26)

What are my options? Many participants were reassured by explanations about voluntariness:

[It is] very reassuring that you’re not locked in to this, that you can stop, and being able to opt out is critical. (29)

### Sentences highlighted as concerning

Of the 215 sentences in the consent form, participants on average highlighted 14 as especially concerning (range: 0–67) (Additional file 1: **Appendix S3**). Sections of the form containing the sentences highlighted as concerning most commonly (i.e.*,* by ≥20% of participants) included (Table 4):

**Table 4:** Sentences commonly highlighted as concerning^a^

Section / Subsection^*^	Sentences Highlighted
**WHAT WILL YOU ASK ME TO DO?**	
We will get some information from your medical records.	
We will use your medical records from time to time to update this information.	
If you agree, we may get information from your mobile health tracker.^*^	
**WHAT WILL YOU DO WITH MY SAMPLE AND INFORMATION?**	
Researchers will have two ways to access the stored materials.	
We will put some information in a **public database** that anyone can look at.	
**WHAT ARE THE RISKS AND HOW WILL I BE PROTECTED?**	
Someone could identify you.	
Your medical records contain information about you and your health. Now or in the future, they could have information you find sensitive.	
Your *mobile tracker* can give clues about your health and lifestyle (such as your activity level), as well as your location.	
[T]here is a risk that someone without permission could get access to the data we have stored about you. Even without identifiers, there is a chance someone could trace it back to you by linking all the data together.	
The stored materials could be used for studies you do not like.^*^	
Your sample and information could be of interest for reasons other than research.^*^	
Because your materials give information about you and your health, they could be of interest to employers, insurers, law enforcement, and others.	
[There are federal laws that protect you from some types of discrimination.] For example, it is illegal for health insurance companies and group health plans to discriminate against people based on genetic information or health conditions. These laws do not protect against discrimination in life insurance, disability insurance, or long-term care insurance.	
Federal laws also make it illegal for most employers to use your genetic information against you. But they do not apply to companies with fewer than 15 employees.	
Your data could be of interest to law enforcement or in a legal case that comes up in your own life. There are federal laws that require us to refuse to give out information that identifies you, even if ordered to by a court or judge, without your okay.	
*[Concluding paragraphs of Risks & Protections section]*	
Politicians could change the laws.	
**WILL I GET THE RESULTS OF STUDIES ON MY SAMPLE AND INFORMATION?**	
Getting results may affect your privacy risks.^*^	
[We will not give information to insurance companies.] But for some insurance (such as long-term care, life, and disability), companies can ask if you have genetic information about yourself or look for it in your medical record. This could hurt your chances to get or keep these types of insurance.	

^a^Sentences highlighted as concerning by 6 (~ 20%) or more participants; see Additional file 1: Appendix S2 for the complete consent form and highlighting counts

^*^Section / Subsection information is provided primarily as a navigational aid for readers; instances where the heading itself was frequently highlighted are denoted with an asterisk

What will you ask me to do? Some participants were concerned about ongoing access to medical records. Of these, some indicated they wanted more logistical details:

[I’m] just curious about how that takes place if, say, you move somewhere and you’re seeing a different medical team. Is that something patients are going to have to be updating with y’all—to let you know, ‘Hey, I have a different person keeping up my medical records’? How are y’all updating the medical records from time to time? What amount of access do they have to the medical records? (03)

How often would they access the medical records, and is that an automatic consent that once you decide to take part, can they go in every week if they want? (27)

Others voiced privacy concerns:

It sounds like you’re signing away your privacy, so to speak. Two years later, you go to the doctor … maybe you finally found out something that, ‘Hey, I don’t want anybody to know about.’ Can this research company just call, ‘Hey, Dr. Smith, I need Joe’s information, blah-blah-blah,’ and the doctors hand it over? I’m concerned about that. (13)

You have the blood—why do you have to have all my personal information? It’s like you asking my life history … That’s a little much. (35)

Another procedure some found concerning was the possibility of obtaining information from mobile health devices:

That might be taking it a step too far for people, because some of those have GPS trackers in it and things like that. Even though you can opt out, the fact that it’s in [the form] is a little iffy. I don’t know if want to be tracked everywhere I go when I’m running, or wherever. Just a strange thing to put in there. (25)

What will you do with my samples and information? The sentence stating that some information would be placed in a publicly-accessible database was particularly concerning. As noted, some participants highlighted this disclosure as reassuring, but many had the opposite reaction:

I don’t want my information on the public database if it’s supposed to be private. It just takes the privacy away when you store it in a public database that anyone can come and look at... Even though it won’t contain any names or other direct identifiers. I mean, where there’s a will there’s a way, in my opinion. (09)

Public database can be hacked easier than a scientific database, so I don’t like that. (16)

What are the risks and how will I be protected? Commonly highlighted sentences in this section included disclosures about:

*Privacy risks*. Statements about access to potentially sensitive information in medical records were particularly concerning:

Your medical records contain information about you and your health. Some things could be alcohol, drug use, mental health, or sexual health. That’s the problem that ... Some things you’ve done in the past. You’re like ‘that was stupid, I was young.’ You don’t want that advertised. (22)

Some participants were concerned by the description of risks due to the optional collection of data from mobile devices:

‘The mobile tracker can give clues about your health and lifestyle such as your activity level, as well as your location.’ I’m a single female that lives alone. Kind of creeps me out that somebody knows where I am. (31)

Sentences describing the prospect of unintended access to information and re-identification were among the most frequently highlighted as concerning. Examples of commonly expressed sentiments include, “Oh, that’s bad. I just felt that sounded bad to me” (07) and “I don’t feel secure at all with that” (20).

#### Objectionable use

Some participants were concerned by the possibility that stored materials could be used for studies they do not like or would rather not support:

My participation will solely be for grabbing a million people and for health care betterment or whatever. I don’t understand what other kind of studies that they might be doing outside of what I think that I’m signing up for. (27)

I may find a few questionable things that I would rather not be part of, and I understand that I won’t necessarily know about it. Now, I do understand that most of it will be beneficial, but there’s always a possibility of something that I would rather not take part of. My information will still be available for that study. That’s something that makes me think about, ‘Well, do I really want to participate or not?’ (23)

#### Non-research use

Sentences describing potential employer, insurer, and law enforcement interest in the stored samples and data were particularly concerning:

That was not the purpose of me becoming a participant in the study. How will they know? It talks about insurance may come and get the information. How are they gonna know to come after *me*? Law enforcers, employers. I’m not identified on this study. You’ve already protected my rights and said that if it’s not for study-related purposes and curing some future disease, why would they even be involved? (20)

I’ve probably watched too many sci-fi movies or whatever, but ‘Your data could be of interest to law enforcement.’ I just immediately go to, what if my hair is planted at the scene of a crime? And now my DNA’s in this database and the police think I’ve committed a crime even if I haven’t. (31)

Although, as noted previously, some participants were reassured by the existence of federal laws against employment discrimination, sentences describing GINA were commonly highlighted as concerning—particularly descriptions of its limitations:

It’s like it’s saying two things. It says it’s illegal for them to do it, but then the laws don’t stop them from doing it. (21)

There are federal laws about preexisting conditions [but] we have no guarantee of that now. That could change any day. I’m no longer working so it doesn’t affect me, but it certainly affects family members that they might not be able to get coverage in the future. (05)

Similarly, although some participants were reassured by the existence of Certificates of Confidentiality, many were not: “To me, it’s kind of like a restraining order, how it’s just a piece of paper—so it doesn’t really protect you.” (12)

#### Changing socio-political landscape

Some were concerned by caveats in the applicability of federal law, including that politicians could change the laws and new risks might arise:

This is just compounded by the fact that this information is going to be there for an unlimited amount of time, and the laws can change, and there’s no telling how it could be affected. (23)

I personally don’t know much about politics and don’t know if it would ever become legal for employers to use genetics. So that just kind of stuck out to me. (01)

Will I get the results of studies on my sample and information? Finally, participants found it concerning that getting individual research results could affect their privacy risks. They were particularly concerned by sentences describing the relationship between receiving individual results and gaps in GINA. This led some to negatively view the value of getting results:

If I have information about something in my DNA that might make me ineligible for life insurance, I am apparently required to disclose it. In this case, knowledge isn’t really power. I’d have information that would only have the capability to work against me and not in my favor, so I don’t like that. (23)

There’s the potential that this is going to prevent me from getting some type of long-term care or disability insurance. There was no benefit to me, but there’s risk to me. Risk versus reward. There’s a lot of risk but very, very little reward. (29)

Others questioned how gaps in GINA would, in fact, be realized:

Getting results may affect your privacy risk, so basically they sayin’ if I get the results, it could put my information out there maybe? I just don’t understand why, if the privacy’s supposed to be protected from the beginning with or without results. (34)

I think that if [the researchers] called me, they found something, I told my physician—I don’t know how the insurance company would get that information. Because I know my physician says everything we discuss and in my record is also private. So even including this, where does insurance come into even getting this information? (20)

### Willingness to participate

After initial review of the consent form (Time 1), 75% of participants rated themselves somewhat to very likely to participate (Table 5). Altruism was a common theme:

I believe it’s important for research to be done to possibly help people in the future or even right now. I think a study is a great thing to do because so many things can be found out and may be used to help somebody. (06)

I like to be able to give what I can in society if it will help other people. Something like this program has a lot of benefits for medical studies, research on diseases, things like that, where it can help the people in the world. (32)

If I can be of help to the future generations, I would be very happy to. I’ve always been in favor of research and I think it’s very important and I think it has been helpful for my generation and so I’m just 100 percent in favor. (19)

Interest in supporting research on specific conditions was another common motivation for participation, particularly given personal or familial experience:

I love the fact that they want to do research on things to cure cancer or diabetes or other diseases ... any kind of chronic disease that they don’t have the cure for. I love the fact that there’s studies that keep on working for it. (07)

My highest reason is because I have a daughter that has a genetic microdeletion. I think any kind of research and study for any kind of ... there’s so many things that people have. Any kind of research towards that would be great. (26)

Among the 25% who rated themselves somewhat to very unlikely to participate, some voiced dissatisfaction regarding compensation:

It’s not that I’m afraid for my information to be out there, I just know the value of that information, and I don’t think that the compensation matches the value. (12)

Concerns about unintended access and use was another common theme …

I know that databases can be hacked. Who knows what the hackers would do with that information? Are they going to try to attach me to somebody who has committed crimes or take my DNA or something like that? (16)…

including by insurance companies in particular:

If entities wanted to find out proof and say, ‘Well, we’re gonna yank your long-term care,’ or ‘We’re not going to offer you long-term care because we know you’re going to be hard to take care of down the road,’ that’s discrimination right there. Until better laws are in place to protect those things, it raises a lot of questions. (16)

After thinking more specifically about what they found reassuring and concerning via the highlighting activity (Time 2), a smaller proportion (66%) of participants rated themselves somewhat to very likely to participate (Table 5). Although half rated their willingness the same as they had at Time 1, one-fourth of participants rated their willingness more negatively by one to four points, and one-fourth rated their willingness one point more positively on the 6-point scale (Additional file 1: **Appendix S3**). Among those who indicated their willingness had decreased, common themes included concerns about unanticipated uses, privacy, risk/benefit ratio, and future risks:

[Decreased from *unlikely* to *very unlikely*] I have now let it marinate more in my mind about my information being used to harm groups of people, and also being used in any kind of legal case against me … I don’t want anyone to build a case against me, even if I didn’t do it—it’s still a risk for anyone, even if you don’t commit crimes. (12)

[Decreased from *somewhat likely* to *unlikely*] I don’t think my information will be as private as they initially tried to sell me on in this study. The only reason I didn’t mark [very unlikely] is because there’s still the possibility of, if I participate, being able to help someone. (20)

[Decreased from *very likely* to *likely*] The more I thought about it, it’s more like I don’t know what’s going to happen in the future, in a few years, how advanced technology might get. Can you protect my data that well if we’re not sure what’s coming up in the next five to ten years? (25)

Among those who indicated their willingness to participate had increased, most felt the benefits outweighed the risks:

[Increased from *unlikely* to *somewhat likely*] That still doesn’t mean I’m feeling all the way comfortable with some of the words in there, but me going through and highlighting allow me to feel like I will be a part of something bigger than me. Going and intentionally looking for things that made me feel secure, it kinda pinpointed the fact that I will be a part of something that will prevent someone from dying from a disease or anything like that. (09)

After considering the values questions (Time 3), the proportion of participants who rated themselves somewhat to very likely to participate remained the same (66%) (Table 5). A large majority (81%) indicated no change and none rated their willingness more negatively, but some (19%) rated their willingness slightly more positively (although still leaving themselves on the same general side of the scale) (Additional file 1: **Appendix S3**). Among those who indicated their willingness to participate had increased, most reported that the questions helped them focus on key personal considerations:

[Increased from *somewhat likely* to *very likely*]: I personally don’t have anything that I’m trying to safeguard with my DNA—[the values questions] made me think through that. The part about talking to your family was a really big piece in the [consent form]. Here, when it’s just in one sentence, I realized that that really does not matter to me at all. Really, the overall good that it can do for the entire population kind of outweighs the potential negatives, I guess. The portion about being used for research or things that you would rather not support, to me personally, it’s kind of out of sight, out of mind. (01)

**Table 5:** Willingness to participate (*n* = 32)

	Time 1: After initial review ^a^	Time 2: After highlighting ^b^	Time 3: After values questions ^c^
	n (%)	n (%)	n (%)
Very unlikely	2 (6)	2 (6)	2 (6)
Unlikely	4 (13)	7 (22)	5 (16)
Somewhat unlikely	2 (6)	2 (6)	4 (13)
Somewhat likely	6 (19)	5 (16)	4 (13)
Likely	9 (28)	8 (25)	6 (19)
Very likely	9 (28)	8 (25)	11 (34)

^a^At Time 1, we asked: “How likely would you be to participate in the Million American Study, on a scale from 1 to 6 where 1 is very unlikely and 6 is very likely?”

^b^At Time 2, we asked: “Now that you’ve had a chance to think about it more, I’m going to ask you again about how likely you would be to participate in the pretend study. Your answer might be the same as before or different. Either is okay”

^c^At Time 3, we asked: “Now that you’ve had a chance to think through these questions, I’m going to ask about likelihood of participating one last time. Your answer might be the same as before or different. Either is okay”

### Helpfulness of values questions

Nearly three-fourths (72%) of participants said the values questions were helpful, describing them as “a good encapsulation of the main areas of concern for people” [15] and an opportunity to “see how you really feel about it, and … give yourself an accurate assessment on whether or not you want to proceed” (09):

This consent form … takes a while to read and to digest. I can tell you after reading it three times, there are things that I missed the first time, and maybe the second time. Because it is so long, there’s critical information in there that someone who would be participating would miss, that they may feel it’s important. So, I think the summary questions do help, because they really hit the high points. (10)

Just breaking down the specific things that could cause concern for you to really look—and to assign the numerical value, too. To say, ‘Yeah, that’s important to me,’ but a three and a five is very different. Of just kind of saying, ‘Is this a big enough issue one way or the other to make the decision?’ (27)

For someone that’s on the fence, it would be a good tool for them to use to think it through, and really think about some of the pros and cons and decide if the potential risk is worth the reward, the knowledge that could be gained from their information. (31)

One-fourth (25%) gave neutral or mixed reviews, saying some people might have made up their minds prior to considering the values questions:

It would depend on the individual because there are some people that are dead hard set, ‘My information is private and I don’t want it out there.’ Then, there’s people like me that are like, ‘It doesn’t bother me. Whatever.’ I’m not sure if [the questions] would help or not. I already had it in my mind … that I am for it, I like it. I guess it would just depend on the individual. (22)

Only one participant said the values questions were not helpful—mostly because they assigned high importance to all the values.

## Discussion

Public, patient, and participant perspectives on biobank and genomic cohort research have been the frequent subject of previous research [22–25]. These studies have oftentimes been conducted using a narrative description of the research endeavor. Our study enriches this literature by connecting the specific, empirically derived wording and organization of a full model consent form with granular qualitative data regarding what members of the general public found especially reassuring or concerning. Further, we developed our model language to meet new U.S. regulatory requirements as well as reflect the rapidly evolving landscape of large-scale precision medicine research. We also crafted a brief set of questions intended to assist prospective participants in considering consent information relative to their own values. Our results provide important insights as well as a foundation for further research.

Consent information our participants most often highlighted as especially reassuring included that the purpose of the biobank was to collect and store materials for studies on health and disease, with the goal of finding new ways to address health problems; the existence and composition of a multidisciplinary oversight committee; a general statement about the importance of participants’ privacy and efforts to protect it, as well as disclosures about basic security safeguards and laws, maintaining data in coded form, and restricting access to direct identifiers; and controlled access to a scientific database, including ethics training and data use agreements.

Information our participants most often highlighted as especially concerning included that data would be made available through a publicly accessible database; the general risk of unintended access to data via triangulation, breach, or hacking; the potential for data to be used for non-research purposes, including by law enforcement and by insurers and employers not covered by GINA; and use of medical record information in general.

Interestingly, our model form contains three sentences that were identified as both reassuring and concerning. Some participants liked that data would be placed in a public database, making it more widely available for potentially beneficial research, while others expressed reservations about privacy and identifiability. Disclosures about federal laws against genetic discrimination reassured some participants but raised questions for others about how employers and insurers could get access to their research data. Finally, information about Certificates of Confidentiality was reassuring to some, while others doubted the protection Certificates could provide.

These findings are generally consistent with others’ research on attitudes toward biobank and genomic cohort research participation. For example, systematic reviews [22–25] indicate that frequently mentioned motivators include a desire to support scientific progress and contribute to the generation of new knowledge and therapies; and perceptions of benefit for self, family, and others. Frequently mentioned reservations include concerns about data privacy and security; the potential for discrimination by the government, employers, and insurers; and the prospect of research that might be contrary to their values. This consistency of findings suggests that the model language we developed, based on our own long-standing programs of research, is effective in meeting new regulatory requirements that information be provided that a reasonable person would want to have about the reasons why one might or might not want to participate in the research (45 CFR 46.116).

A main purpose of our study was to explore participants’ positive and negative reactions to the specific way this information was communicated by our consent form (as captured by the sentence-level highlighting activity). We believe the data we collected about hypothetical willingness to participate is an indicator of the net impact, i.e.*,* each participant’s own assessment of the combined effect of details they found reassuring and concerning. The proportion of our participants who responded favorably was broadly similar to the 60–68% found in large-scale surveys of the U.S. population [26–28]. Across these surveys, willingness to participate was associated with demographic factors (e.g.*,* self-reported race, income, education, age, religiosity) and biobank design features (e.g.*,* access to individual research results, compensation, study burden), as well as degree of support for the basic purpose of the research and degree of concern about issues such as privacy and morally objectionable research.

Although our study was not designed to quantitatively assess such factors, our participants’ willingness to take part decreased after the highlighting activity (compared to after their initial reading of the form). Their willingness did not notably change upon considering the values questions. This could be an artifact of our study design, i.e.*,* that the highlighting activity itself (unlikely to be replicated in an actual consent process) served to engage participants in carefully considering the information in the form. Regardless, the values questions we devised were not intended to change participation decisions per se, but rather to be as neutral as possible and based on evidence generated in empirical research with a diverse group of prominent experts and scholars in the areas of ethics, genome research, health law, historically-disadvantaged populations, informatics, and participant-centric perspectives, as well as government officials and human subjects protections leaders [5, 15, 16]. Our goal was to create a simple tool that would prompt prospective participants to think through their personal values and what key consent disclosures might mean for their individual context.

The results of our study are subject to some limitations. We used a hypothetical design to gather preliminary input. In an actual consent setting, prospective participants may consider or weigh factors differently; similarly, the utility ascribed to a decision aid (such as our values questions) under such circumstances may vary. Although our sample was demographically diverse, it was small and constrained to one geographic location. Future research should be undertaken to assess perspectives in other regions and populations, including larger sample sizes to enable statistical comparisons.

Additionally, the focus of this study was on the content, organization, and simplification of empirically derived model consent language. Further research is needed to determine whether the length of the resulting form could be reduced while still meeting regulatory requirements and best practice guidelines, as well as clearly conveying the information a reasonable person would want to have in order to make an informed decision about whether to participate.

## Conclusions

Our study contributes to efforts to improve informed consent for genomic and precision medicine research, much of which entails the collection and analysis of multidimensional data and value-laden risks and benefits for participants, families, and communities. In addition to providing model consent language and preliminary reactions from members of the general public, our findings suggest that consent interventions that facilitate prospective participants’ closer engagement with the information, such as decision aids [19, 20], may result in participation decisions that align more closely with their values. This is an important area for detailed exploration in future research. For example, the set of values questions we devised merit further research on both content and effect on participant decision-making. It is possible that real-life implementation of such a tool could positively influence participants’ perceived understanding, satisfaction with their decision, trust in research, and long-term retention and engagement in biobanking and precision medicine research [29–32].

## Supplementary information

**Additional file 1.** S1: Complete consent form with readability characteristics. S2: Complete consent form with highlight counts and regulatory justification. S3: Figures 1, 2a-b, and 3.

## Data Availability

The datasets generated and analyzed in this study are not publicly available due to privacy and confidentiality considerations, but are available upon reasonable request from qualified researchers conducting IRB-approved studies that fall within the scope of the study purpose and data use described to interviewees at the time of participation.

## Abbreviations

CFR: United States Code of Federal Regulations
US: United States
