# Supplementary material for: Developing model biobanking consent language: what matters to prospective participants?
Source: BMC Med Res Methodol. 2020 May 15;20:119. doi: 10.1186/s12874-020-01001-2 (PMC7227271; doi:10.1186/s12874-020-01001-2)

SUPPLEMENTAL INFORMATION

Developing model biobanking consent language: What matters to prospective participants?

Laura M. Beskow, MPH, PhD[1]; Catherine M. Hammack-Aviran, MA, JD[1]; Kathleen M. Brelsford, PhD, MPH[1]

[1]Center for Biomedical Ethics and Society, Vanderbilt University Medical Center, Nashville, Tennessee, USA

Corresponding author: Laura M. Beskow, MPH, PhD, Center for Biomedical Ethics and Society, Vanderbilt University Medical Center, Nashville, TN, USA 37203; telephone: 615-936-2686; email: laura.m.beskow@vanderbilt.edu

CONTENTS:

- S1: Complete consent form with readability characteristics
- S2: Complete consent form with highlight counts and regulatory justification
- S3: Figures 1, 2a-b, and 3

S1. CONSENT TO PARTICIPATE IN THE MILLION AMERICAN STUDY

We are asking you to join a research project called the Million American Study. Taking part in this project is your choice. This form explains the project so you can decide. Please read it carefully and take all the time you need. Feel free to talk it over with your family, friends, and doctor. Be sure to ask any questions, now or later. There is a list of people you can ask at the end of this form. If you decide to join, we will give you a copy of this form to keep.

WHY IS THIS PROJECT BEING DONE?

The purpose of the Million American Study is to collect and store blood and health information. Researchers will use the stored materials in future studies on health and disease. Through such studies, they hope to find new ways to detect, treat, and maybe prevent or cure health problems.

We hope at least 1 million people in the U.S. will take part.

WHO IS DOING THIS PROJECT?

The federal government is funding the Million American Study. Dr. Terry Lee at Springfield University is leading the project.

There is a Steering Committee to watch over the project and make sure we are doing things the right way. This includes researchers, doctors, lawyers, ethics experts, and government officials. It also includes patients and members of the public to help speak for people who take part.

WHAT WILL YOU ASK ME TO DO?

If you decide to join the Million American Study, here is what will happen:

- We will get a blood sample from you.

  We will use a needle to draw about 3 tablespoons of blood from your arm.

  Blood contains many things related to our health. For example, it has fats, minerals, and chemicals that are naturally in our bodies. It also has markers for things we get from our environment, such as from our food, water, and air.

  Blood also contains DNA. Your DNA contains your unique genetic code. This code is the blueprint for building the proteins that make the cells in our bodies work.

- We will ask for information about you and your health.

  We will ask you for some basic information, such as your name, date of birth, gender, and race. We will also ask about your health habits, the places you live and work, and your family's health history. We will contact you about once a year to ask for updates.

- We will get some information from your medical records.

  For instance, we will get information about your test results, medical procedures, images (such as X-rays), and medicines you take. We will use your medical records from time to time to update this information. We will do this for as long as you are taking part in the Million American Study.

- If you agree, we may get information from your mobile health tracker.

  Some people use mobile apps or devices to keep an eye on their health. For example, people use them to track their heart rate or count the steps they take in a day.

  If you use one of these trackers, you may be able share information from it with the Million American Study. Signing up for this part of the project is optional. You can still join even if you say 'no' to this part.

- We may ask if you want to hear about other research.

  We may contact you later with offers to take part in other research. There will be a new consent form just for these other studies, so you can choose then to take part or not. You can say 'no' and still be in the Million American Study.

WHAT WILL YOU DO WITH MY SAMPLE AND INFORMATION?

- We will store all the samples and information in a central place.

  We will store your sample and information, along with those from all the other people who take part, at a secure location in Norfolk, Virginia. There will be many layers and kinds of safeguards to help keep the materials safe.

- We will not give researchers anything that directly identifies you.

  When we store the materials, we will remove names and other identifiers. We will replace them with code numbers. There will be a master list linking the code numbers to names, but we will keep it separate from the samples and information. Only a few project staff can see the list, and they sign a pledge to keep it secret.

  People who study the samples and information will not know who you are. We will give them materials labeled with only the code numbers.

- We will let many kinds of researchers use the stored materials.

  Researchers who study the materials will be from many places. They could be from academic or other non-profit institutions, the government, or commercial companies (such as drug companies). Some researchers will be from the U.S., some may be from other countries.

- Researchers will do many kinds of studies to learn about health and disease.

  Researchers will use the stored materials for lots of different studies. They will come up with topics over time, so we do not know right now the exact things they will look at. In general, they will be trying to find out more about:

  - What causes people to be healthy or have a disease?
  - Why do diseases and treatments affect people differently?
  - How do basic biology, lifestyle, and environment work together to affect health?

  Some of these studies may be about how genes affect health. Some may include DNA sequencing to learn the order of the building blocks that make up each person's genetic code. Some of the studies may lead to new products, such as drugs or tests for diseases.

  We will add the results of the studies that are done to the information we have stored so other researchers can learn from it.

- Researchers will have two ways to access the stored materials.

  1) We will put some information in a **public database** that anyone can look at.

     This database will not contain names or other direct identifiers. Further, it will not allow anyone to see information about just one person. It will only show information at a group level (for instance, for the group of people in the Million American Study who have heart disease).

  2) We will also make a **scientific database**. Like the public one, this database will not contain names or other direct identifiers. Researchers who use it will still be looking at large groups of people. But they will be able to see individual-level information for each person in the group. We may also allow them to analyze part of the samples.

     Access to the scientific database will be strictly controlled. Researchers who want to use it must first complete ethics training. Then they must apply to do their specific study. An Access Committee will review each request. If a study is approved, the researcher must sign a pledge to use the materials only for that study. They also promise to keep the materials secure and not try to figure out who you are.

HOW LONG WILL YOU KEEP MY SAMPLE AND INFORMATION?

There is no limit on the length of time we will store your sample and information. We may go on letting researchers study them for as long as they are useful, unless you ask us to stop.

If your sample gets used up, we may ask you to give another one. You can say 'no' and still be in the Million American Study.

ARE THERE ANY BENEFITS?

You should not expect to get direct health benefits. The main reason you may want to join is to help researchers learn things that could improve health care for people in the future.

WHAT ARE THE RISKS AND HOW WILL I BE PROTECTED?

We believe the risks of taking part in the Million American Study are low, but they are not zero. People have their own life situations and might view the risks differently. Here is some information to help you think about how they might matter to you:

- Someone could identify you.

  The main risk in taking part in the Million American Study is to your privacy. We will collect, store, and share many types of data about you:

  - Your *blood sample* contains your DNA. Your DNA is unique to you. It also holds information that may suggest some possibilities for your current and future health.
  - Your *medical records* contain information about you and your health. Now or in the future, they could have information you find sensitive. For example, they could have things about alcohol or drug use, mental health, and sexual health.
  - Your *mobile tracker* can give clues about your health and lifestyle (such as your activity level), as well as your location.

  Your privacy is very important to us and we will make every effort to protect it. We will keep everything in a secure place and label it only with a code. We will not give out anything that tells who you are. Nobody will know just from looking at the databases that the information belongs to you.

  Even so, there is a risk that someone without permission could get access to the data we have stored about you. Even without identifiers, there is a chance someone could trace it back to you by linking all the data together.

We will follow federal rules designed to make sure only the right people see your data. These include limiting and tracking who has access, as well as passwords, encryption, and other safeguards. We will tell you if someone sees the data who was not supposed to.

- <u>The stored materials could be used for studies you do not like</u>.

  Many kinds of researchers will use the materials for many kinds of studies. We will not notify you every time your sample and information are used. This means they could be used by a type of researcher or for a topic you would rather not support. For instance, you might object to some aspect of a study based on your personal beliefs.

  There could also be studies that have meaning for your community. This is because we will give researchers basic facts such as your race, ethnicity, and gender. Researchers use these to learn about factors that lead to health or disease in different groups of people. Such findings could one day help people of the same race, ethnicity, or gender as you. However, they could also be misused to support harmful ideas about groups.

  The goal of the Million American Study is to make discoveries that improve health for everyone. The Steering Committee and the Access Committee have the duty to make sure the studies that are done help reach this goal.

  You can learn about the studies being done on the Million American Study web site [URL]. We will post updates about studies that have been approved and summaries of research findings. We will also post reports on uses of the public database. You can always reach us with any questions or concerns. You have the right to leave the project at any time (see the part below, “What are my options?”).

- <u>Your sample and information could be of interest for reasons other than research</u>.

  Because your materials give information about you and your health, they could be of interest to employers, insurers, law enforcement, and others. There are laws to stop those who want to use your data for things other than research, but they are not foolproof.

  - There are federal laws that protect you from some types of discrimination. For example, it is illegal for **health insurance** companies and group health plans to discriminate against people based on genetic information or health conditions. These laws do *not* protect against discrimination in life insurance, disability insurance, or long-term care insurance.

  - Federal laws also make it illegal for most **employers** to use your genetic information against you. But they do *not* apply to companies with fewer than 15 employees.

  - Your data could be of interest to **law enforcement** or in a legal case that comes up in your own life. There are federal laws that require us to refuse to give out

information that identifies you, even if ordered to by a court or judge, without your okay. Still, we must follow laws that require us to report certain things to **state officials**. These include serious diseases that spread easily to other people, domestic violence or abuse, and threats to harm yourself or others. If we share information that identifies you outside the Million American Study, it may no longer be protected by the federal privacy law called 'HIPAA.' But it may be covered by other privacy rules or agreements.

The Million American Study will only allow people to use the stored materials for research purposes. The main way employers, insurers, law enforcement, or others might be able to get your data is from you, if you receive your own research results (see the part below, "Will I get the results?").

- The stored materials could suggest information about your family.

  You and your family may have many health-related things in common. For example, you might live in the same place or eat the same kinds of food. You inherit your DNA from your parents and pass it on to your kids. So, your genetic information, as well as your medical record, could give clues about who your family members are, what genes you may share with them, and what their health might be like.

  This means the risks we describe in this form could extend in some ways to your family. Some of them may feel differently about this than you do. You may want to talk with them about your decision to join the Million American Study.

The federal laws we described protect you no matter where you live. Some states have laws that might provide more protections (but never less).

The information above outlines the main risks and protections right now. However, researchers will study the stored materials long into the future. Things could happen over time that change the risks. For instance, technical advances could make it easier to re-identify research data. Politicians could change the laws. New risks might come up that we cannot predict right now.

We will tell you if we learn of anything that might change your decision to take part.

ARE THERE ANY COSTS OR PAYMENTS?

There are no costs to you or your insurance. We will give you a $25 gift card to thank you for the time it takes you to sign up.

Some research may lead to new products, such as drugs or tests for diseases. If so, researchers and others who own these products may profit from their sale. There are no plans to share any profits with you. Development of new products usually relies on the study of samples and information from hundreds or thousands of people, not from only one person.

WILL I GET THE RESULTS OF STUDIES ON MY SAMPLE AND INFORMATION?

You should not expect to get individual results from studies done through the Million American Study. Researchers must study materials from many people over many years before they can know if the results have meaning.

There is a small chance that researchers could find something that might be very important to your health or medical care right now. At the end of this form, you can tell us whether you want us to try to contact you if this happens. Here is some information to help you decide:

- Research is not the same as medical care.

  If we do not contact you with any results, do not assume that means everything is okay. Talk to your doctor if you have any questions or concerns about your health.

  If we do contact you, we will give you some basic information about the results. It will be up to you whether to share the results with your doctor. The Million American Study will not cover the cost of any health care you might decide to get based on your results.

- Results could be upsetting or surprising.

  If we contact you with results, they could be helpful to your health or medical care. But they may also make you worried, anxious, or upset. For example, depending on your resources and health care in your area, it could be hard to find and get access to the right kind of follow-up care. The results could have information you did not expect, such as surprises about your family tree.

  Your results might also hint at some possibilities for your family members' current and future health. Some of them may feel differently about getting the results than you do. You may want to talk with them before getting or sharing your results.

- Getting results may affect your privacy risks.

  The Million American Study will protect your research results the same as we protect all your data. But if you choose to get any results we offer, you will not be able to shield the information the way we can. For instance:

  - If you share your results with your doctor, they will become part of your medical record. The protections for your medical record are strong, but not perfect.
  - We will not give information to insurance companies. But for some insurance (such as long-term care, life, and disability), companies can ask if you have genetic information about yourself or look for it in your medical record. This could hurt your chances to get or keep these types of insurance.

WHAT ARE MY OPTIONS?

Taking part in the Million American Study is your choice. You can choose to join or not. If you decide to join, you can change your mind at any time. Just let us know. If we have given out some of your materials for study, we cannot get them back. But we will send you a form so you can tell us what to do with your information and any of your sample that are left.

If you decide not to participate, now or in the future, it will not affect your medical care.

WHO CAN ANSWER MY QUESTIONS?

<table>
<tr><th>If you have questions or concerns about:</th><th>Please contact:</th></tr>
<tr><td>Your health</td><td>Your doctor</td></tr>
<tr><td>The Million American Study</td><td rowspan="3">Dr. Terry Lee
Springfield University
Terry.Lee@xxx.edu
555-864-2153</td></tr>
<tr><td>Questions or concerns about studies or data uses</td></tr>
<tr><td>Withdrawing from the Million American Study</td></tr>
<tr><td>Your rights as a research participant</td><td>Institutional Review Board
Springfield University
IRB@xxx.edu
555-171-1086</td></tr>
</table>

READABILITY CHARACTERISTICS:

Flesch-Kincaid grade level	7.2
Flesch-Kincaid reading ease<br>*(100-point scale; the higher the score, the easier it is to understand)*	68.6
Passive sentences<br>*(active sentences are easier to understand)*	8.1%

S2. COMPLETE CONSENT FORM WITH HIGHLIGHT COUNTS AND REGULATORY JUSTIFICATION

KEY:	
	6+ participants (~20%+) highlighted as **reassuring**
	6+ participants (~20%+) highlighted as **concerning**

BLUE = Common Rule, requirement for broad consent
PURPLE = Common Rule, general requirement for all consent
GOLD = Common Rule, requirement for other than broad consent
PINK = HIPAA Privacy Rule

Line Num	Sentence	# marked as Reassuring	# marked as Concerning	Regulatory Requirement	Req. met by Line Nums
0	**[TITLE] CONSENT TO PARTICIPATE IN THE MILLION AMERICAN STUDY**	0	0		
1	We are asking you to join a research project called the Million American Study.	0	0	§ 46.116(b)(1)<br>§ 164.508(c)(1)(iv)	1<br>1-94
2	Taking part in this project is your choice.	3	0	§ 46.116(b)(8)	2; 208-214
3	This form explains the project so you can decide.	0	0	§ 46.116(a)(2)	3-7
4	Please read it carefully and take all the time you need.	0	0	§ 46.116(a)(4)	4-7
5	Feel free to talk it over with your family, friends, and doctor.	4	0		
6	Be sure to ask any questions, now or later.	3	1		
7	There is a list of people you can ask at the end of this form.	3	1		
8	If you decide to join, we will give you a copy of this form to keep.	0	0	§ 164.508(c)(4)	8
9	**WHY IS THIS PROJECT BEING DONE?**	2	0	§ 46.116(b)(1)	9-12
10	The purpose of the Million American Study is to collect and store blood and health information.	5	2		
11	Researchers will use the stored materials in future studies on health and disease.	11	0		

Line Num	Sentence	# marked as Reassuring	# marked as Concerning	Regulatory Requirement	Req. met by Line Nums
12	Through such studies, they hope to find new ways to detect, treat, and maybe prevent or cure health problems.	19	0		
13	We hope at least 1 million people in the U.S. will take part.	4	0	§ 46.116(c)(6)	13
14	**WHO IS DOING THIS PROJECT?**	0	0		
15	The federal government is funding the Million American Study.	2	3	§ 164.508(c)(1)(ii)	15-16
16	Dr. Terry Lee at Springfield University is leading the project.	1	0		
17	There is a Steering Committee to watch over the project and make sure we are doing things the right way.	14	0		
18	This includes researchers, doctors, lawyers, ethics experts, and government officials.	14	1		
19	It also includes patients and members of the public to help speak for people who take part.	9	2		
20	**WHAT WILL YOU ASK ME TO DO?**	1	0	§ 46.116(b)(1)	20-89
21	If you decide to join the Million American Study, here is what will happen:	1	0	§ 164.508(c)(1)(i)	21-47; 104-111
22	· <u>We will get a blood sample from you</u>.	4	0	§ 46.116(d)(3)	22-43; 59-92
23	We will use a needle to draw about 3 tablespoons of blood from your arm.	6	1		
24	Blood contains many things related to our health.	3	1		
25	For example, it has fats, minerals, and chemicals that are naturally in our bodies.	3	1		
26	It also has markers for things we get from our environment, such as from our food, water, and air.	3	1		
27	Blood also contains DNA.	4	1		
28	Your DNA contains your unique genetic code.	3	1		

Line Num	Sentence	# marked as Reassuring	# marked as Concerning	Regulatory Requirement	Req. met by Line Nums
29	This code is the blueprint for building the proteins that make the cells in our bodies work.	3	2		
30	· We will ask for information about you and your health.	4	1		
31	We will ask you for some basic information, such as your name, date of birth, gender, and race.	3	2		
32	We will also ask about your health habits, the places you live and work, and your family's health history.	3	2		
33	We will contact you about once a year to ask for updates.	5	2	§ 46.116(b)(1)	33; 37; 91-92; 165
34	· We will get some information from your medical records.	4	5		
35	For instance, we will get information about your test results, medical procedures, images (such as X-rays), and medicines you take.	0	4		
36	We will use your medical records from time to time to update this information.	0	7		
37	We will do this for as long as you are taking part in the Million American Study.	1	3	§ 46.116(b)(1)	33; 37; 91-92; 165
38	· If you agree, we may get information from your mobile health tracker.	3	7		
39	Some people use mobile apps or devices to keep an eye on their health.	0	2		
40	For example, people use them to track their heart rate or count the steps they take in a day.	0	2		
41	If you use one of these trackers, you may be able share information from it with the Million American Study.	0	3		
42	Signing up for this part of the project is optional.	2	2		
43	You can still join even if you say 'no' to this part.	1	3		
44	· We may ask if you want to hear about other research.	5	1		
45	We may contact you later with offers to take part in other research.	2	0		

Line Num	Sentence	# marked as Reassuring	# marked as Concerning	Regulatory Requirement	Req. met by Line Nums
46	There will be a new consent form just for these other studies, so you can choose then to take part or not.	4	0		
47	You can say ‘no’ and still be in the Million American Study.	1	1		
48	**WHAT WILL YOU DO WITH MY SAMPLE AND INFORMATION?**	1	0		
49	· <u>We will store all the samples and information in a central place</u>.	7	0		
50	We will store your sample and information, along with those from all the other people who take part, at a secure location in Norfolk, Virginia.	7	2		
51	There will be many layers and kinds of safeguards to help keep the materials safe.	15	3		
52	· <u>We will not give researchers anything that directly identifies you</u>.	14	0	§ 46.116(b)(9)(i)	52-58; 76; 80; 114-115
53	When we store the materials, we will remove names and other identifiers.	14	0	§ 46.116(b)(5)	53-58; 76-78; 80-89; 112-120; 147-152
54	We will replace them with code numbers.	10	1		
55	There will be a master list linking the code numbers to names, but we will keep it separate from the samples and information.	4	2		
56	Only a few project staff can see the list, and they sign a pledge to keep it secret.	7	5		
57	People who study the samples and information will not know who you are.	10	0		
58	We will give them materials labeled with only the code numbers.	6	0		
59	· <u>We will let many kinds of researchers use the stored materials</u>.	3	3	§ 46.116(d)(3)<br>§ 46.116(b)(9)(i)<br>§ 164.508(c)(1) (iii)	22-43; 59-92<br>59-92<br>59-89
60	Researchers who study the materials will be from many places.	2	2	§ 46.116(c)(7)	60-61; 174-177
61	They could be from academic or other non-profit institutions, the government, or commercial companies (such as drug companies).	2	3		

Line Num	Sentence	# marked as Reassuring	# marked as Concerning	Regulatory Requirement	Req. met by Line Nums
62	Some researchers will be from the U.S., some may be from other countries.	2	4		
63	· Researchers will do many kinds of studies to learn about health and disease.	6	0	§ 46.116(d)(2)	63-89; 121-137
64	Researchers will use the stored materials for lots of different studies.	3	0		
65	They will come up with topics over time, so we do not know right now the exact things they will look at.	3	3		
66	In general, they will be trying to find out more about:	3	1		
67	- What causes people to be healthy or have a disease?	7	0		
68	- Why do diseases and treatments affect people differently?	7	0		
69	- How do basic biology, lifestyle, and environment work together to affect health?	8	0		
70	Some of these studies may be about how genes affect health.	4	0	§ 46.116(c)(9)	70-71
71	Some may include DNA sequencing to learn the order of the building blocks that make up each person's genetic code.	4	0		
72	Some of the studies may lead to new products, such as drugs or tests for diseases.	8	0		
73	We will add the results of the studies that are done to the information we have stored so other researchers can learn from it.	5	0		
74	· Researchers will have two ways to access the stored materials.	2	0		
75	1) We will put some information in a **public database** that anyone can look at.	7	10		
76	This database will not contain names or other direct identifiers.	7	0	§ 46.116(b)(5)<br>§ 46.116(b)(9)(i)	53-58; 76-78; 80-89; 112-120; 147-152<br>52-58; 76; 80; 114-115
77	Further, it will not allow anyone to see information about just one person.	6	0		

Line Num	Sentence	# marked as Reassuring	# marked as Concerning	Regulatory Requirement	Req. met by Line Nums
78	It will only show information at a group level (for instance, for the group of people in the Million American Study who have heart disease).	7	0		
79	2) We will also make a **scientific database**.	10	0		
80	Like the public one, this database will not contain names or other direct identifiers.	6	0	§ 46.116(b)(5)<br>§ 46.116(b)(9)(i)	53-58; 76-78; 80-89; 112-120; 147-152<br>52-58; 76; 80; 114-115
81	Researchers who use it will still be looking at large groups of people.	4	0		
82	But they will be able to see individual-level information for each person in the group.	4	0		
83	We may also allow them to analyze part of the samples.	2	0		
84	Access to the scientific database will be strictly controlled.	12	0		
85	Researchers who want to use it must first complete ethics training.	12	1		
86	Then they must apply to do their specific study.	8	0		
87	An Access Committee will review each request.	8	0		
88	If a study is approved, the researcher must sign a pledge to use the materials only for that study.	11	3		
89	They also promise to keep the materials secure and not try to figure out who you are.	9	4		
90	**HOW LONG WILL YOU KEEP MY SAMPLE AND INFORMATION?**	0	0		
91	There is no limit on the length of time we will store your sample and information.	2	2	§ 46.116(d)(4)<br>§ 46.116(b)(1)<br>§ 164.508(c)(1)(v)	91-92; 165<br>33; 37; 91-92; 165<br>91-92
92	We may go on letting researchers study them for as long as they are useful, unless you ask us to stop.	3	2		

Line Num	Sentence	# marked as Reassuring	# marked as Concerning	Regulatory Requirement	Req. met by Line Nums
93	If your sample gets used up, we may ask you to give another one.	1	5		
94	You can say 'no' and still be in the Million American Study.	2	4		
95	**ARE THERE ANY BENEFITS?**	0	1		
96	You should not expect to get direct health benefits.	1	2	§ 46.116(b)(3)	96-97
97	The main reason you may want to join is to help researchers learn things that could improve health care for people in the future.	6	2		
98	**WHAT ARE THE RISKS AND HOW WILL I BE PROTECTED?**	2	0		
99	We believe the risks of taking part in the Million American Study are low, but they are not zero.	4	1	§ 46.116(b)(2)	99-170; 190-206
100	People have their own life situations and might view the risks differently.	2	0		
101	Here is some information to help you think about how they might matter to you:	1	0		
102	· Someone could identify you.	2	4		
103	The main risk in taking part in the Million American Study is to your privacy.	2	1		
104	We will collect, store, and share many types of data about you:	2	3	§ 164.508(c)(1)(i)	21-47; 104-111
105	- Your *blood sample* contains your DNA.	2	2		
106	Your DNA is unique to you.	2	1		
107	It also holds information that may suggest some possibilities for your current and future health.	2	1		
108	- Your *medical records* contain information about you and your health.	1	8		
109	Now or in the future, they could have information you find sensitive.	1	6		

Line Num	Sentence	# marked as Reassuring	# marked as Concerning	Regulatory Requirement	Req. met by Line Nums
110	For example, they could have things about alcohol or drug use, mental health, and sexual health.	1	5		
111	- Your *mobile tracker* can give clues about your health and lifestyle (such as your activity level), as well as your location.	1	7		
112	Your privacy is very important to us and we will make every effort to protect it.	18	1	§ 46.116(b)(5)	53-58; 76-78; 80-89; 112-120; 147-152
113	We will keep everything in a secure place and label it only with a code.	16	0		
114	We will not give out anything that tells who you are.	13	1	§ 46.116(b)(9)(i)	52-58; 76; 80; 114-115
115	Nobody will know just from looking at the databases that the information belongs to you.	13	0		
116	Even so, there is a risk that someone without permission could get access to the data we have stored about you.	2	12		
117	Even without identifiers, there is a chance someone could trace it back to you by linking all the data together.	2	13		
118	We will follow federal rules designed to make sure only the right people see your data.	12	1		
119	These include limiting and tracking who has access, as well as passwords, encryption, and other safeguards.	11	1		
120	We will tell you if someone sees the data who was not supposed to.	9	4		
121	· The stored materials could be used for studies you do not like.	4	7	§ 46.116(d)(2)<br>§ 46.116(d)(5)	63-89; 121-137<br>121-137
122	Many kinds of researchers will use the materials for many kinds of studies.	3	2		
123	We will not notify you every time your sample and information are used.	3	3		
124	This means they could be used by a type of researcher or for a topic you would rather not support.	3	2		
125	For instance, you might object to some aspect of a study based on your personal beliefs.	3	3		
126	There could also be studies that have meaning for your community.	2	1		

Line Num	Sentence	# marked as Reassuring	# marked as Concerning	Regulatory Requirement	Req. met by Line Nums
127	This is because we will give researchers basic facts such as your race, ethnicity, and gender.	2	2		
128	Researchers use these to learn about factors that lead to health or disease in different groups of people.	2	1		
129	Such findings could one day help people of the same race, ethnicity, or gender as you.	2	1		
130	However, they could also be misused to support harmful ideas about groups.	2	5		
131	The goal of the Million American Study is to make discoveries that improve health for everyone.	9	2		
132	The Steering Committee and the Access Committee have the duty to make sure the studies that are done help reach this goal.	4	1		
133	You can learn about the studies being done on the Million American Study web site [URL].	6	1		
134	We will post updates about studies that have been approved and summaries of research findings.	4	1		
135	We will also post reports on uses of the public database.	3	2		
136	You can always reach us with any questions or concerns.	4	1		
137	You have the right to leave the project at any time (see the part below, “What are my options?”).	9	1		
138	· Your sample and information could be of interest for reasons other than research.	2	10		
139	Because your materials give information about you and your health, they could be of interest to employers, insurers, law enforcement, and others.	1	7		
140	There are laws to stop those who want to use your data for things other than research, but they are not foolproof.	3	5		
141	- There are federal laws that protect you from some types of discrimination.	5	4		
142	For example, it is illegal for **health insurance** companies and group health plans to discriminate against people based on genetic information or health conditions.	3	6		

Line Num	Sentence	# marked as Reassuring	# marked as Concerning	Regulatory Requirement	Req. met by Line Nums
143	These laws do *not* protect against discrimination in life insurance, disability insurance, or long-term care insurance.	4	6		
144	- Federal laws also make it illegal for most **employers** to use your genetic information against you.	7	7		
145	But they do *not* apply to companies with fewer than 15 employees.	2	8		
146	- Your data could be of interest to **law enforcement** or in a legal case that comes up in your own life.	5	8		
147	There are federal laws that require us to refuse to give out information that identifies you, even if ordered to by a court or judge, without your okay.	6	7	§ 46.116(b)(5)	53-58; 76-78; 80-89; 112-120; 147-152
148	Still, we must follow laws that require us to report certain things to **state officials**.	7	4		
149	These include serious diseases that spread easily to other people, domestic violence or abuse, and threats to harm yourself or others.	4	3		
150	If we share information that identifies you outside the Million American Study, it may no longer be protected by the federal privacy law called 'HIPAA.'	3	5	§ 164.508(c)(2)(iii)	150-153
151	But it may be covered by other privacy rules or agreements.	1	2		
152	The Million American Study will only allow people to use the stored materials for research purposes.	5	1		
153	The main way employers, insurers, law enforcement, or others might be able to get your data is from you, if you receive your own research results (see the part below, "Will I get the results?").	1	3		
154	· The stored materials could suggest information about your family.	6	0		
155	You and your family may have many health-related things in common.	2	0		
156	For example, you might live in the same place or eat the same kinds of food.	2	0		
157	You inherit your DNA from your parents and pass it on to your kids.	2	0		

Line Num	Sentence	# marked as Reassuring	# marked as Concerning	Regulatory Requirement	Req. met by Line Nums
158	So, your genetic information, as well as your medical record, could give clues about who your family members are, what genes you may share with them, and what their health might be like.	4	2		
159	This means the risks we describe in this form could extend in some ways to your family.	2	4		
160	Some of them may feel differently about this than you do.	2	1		
161	You may want to talk with them about your decision to join the Million American Study.	2	0		
162	The federal laws we described protect you no matter where you live.	6	1		
163	Some states have laws that might provide more protections (but never less).	4	0		
164	The information above outlines the main risks and protections right now.	2	3	§ 46.116(c)(1)	164-170
165	However, researchers will study the stored materials long into the future.	2	2	§ 46.116(d)(4)<br>§ 46.116(b)(1)	91-92; 165<br>33; 37; 91-92; 165
166	Things could happen over time that change the risks.	2	4		
167	For instance, technical advances could may make it easier to re-identify research data.	2	5		
168	Politicians could change the laws.	2	6		
169	New risks might come up that we cannot predict right now.	3	5		
170	We will tell you if we learn of anything that might change your decision to take part.	6	0	§ 46.116(c)(5)	170
171	**ARE THERE ANY COSTS OR PAYMENTS?**	0	1		
172	There are no costs to you or your insurance.	4	1	§ 46.116(c)(3)	172
173	We will give you a $25 gift card to thank you for the time it takes you to sign up.	4	3		
174	Some research may lead to new products, such as drugs or tests for diseases.	6	1	§ 46.116(c)(7)	60-61; 174-177

Line Num	Sentence	# marked as Reassuring	# marked as Concerning	Regulatory Requirement	Req. met by Line Nums
175	If so, researchers and others who own these products may profit from their sale.	1	3		
176	There are no plans to share any profits with you.	2	3		
177	Development of new products usually relies on the study of samples and information from hundreds or thousands of people, not from only one person.	0	0		
178	**WILL I GET THE RESULTS OF STUDIES ON MY SAMPLE AND INFORMATION?**	0	0		
179	You should not expect to get individual results from studies done through the Million American Study.	2	2	§ 46.116(d)(6)<br>§ 46.116(c)(8)	179-182<br>179-182
180	Researchers must study materials from many people over many years before they can know if the results have meaning.	0	0		
181	There is a small chance that researchers could find something that might be very important to your health or medical care right now.	6	0		
182	At the end of this form, you can tell us whether you want us to try to contact you if this happens.	7	0		
183	Here is some information to help you decide:	2	0		
184	· Research is not the same as medical care.	4	1		
185	If we do not contact you with any results, do not assume that means everything is okay.	1	2		
186	Talk to your doctor if you have any questions or concerns about your health.	1	1		
187	If we do contact you, we will give you some basic information about the results.	4	1		
188	It will be up to you whether to share the results with your doctor.	4	1		
189	The Million American Study will not cover the cost of any health care you might decide to get based on your results.	1	0	§ 46.116(b)(6)	173-177; 189
190	· Results could be upsetting or surprising.	4	3	§ 46.116(b)(2)	99-170; 190-206

Line Num	Sentence	# marked as Reassuring	# marked as Concerning	Regulatory Requirement	Req. met by Line Nums
191	If we contact you with results, they could be helpful to your health or medical care.	3	1		
192	But they may also make you worried, anxious, or upset.	1	2		
193	For example, depending on your resources and health care in your area, it could be hard to find and get access to the right kind of follow-up care.	1	1		
194	The results could have information you did not expect, such as surprises about your family tree.	1	1		
195	Your results might also hint at some possibilities for your family members' current and future health.	3	2		
196	Some of them may feel differently about getting the results than you do.	2	2		
197	You may want to talk with them before getting or sharing your results.	2	2		
198	· Getting results may affect your privacy risks.	4	6		
199	The Million American Study will protect your research results the same as we protect all your data.	4	2		
200	But if you choose to get any results we offer, you will not be able to shield the information the way we can.	1	2		
201	For instance:	1	0		
202	- If you share your results with your doctor, they will become part of your medical record.	3	4		
203	The protections for your medical record are strong, but not perfect.	3	4		
204	- We will not give information to insurance companies.	9	5		
205	But for some insurance (such as long-term care, life, and disability), companies can ask if you have genetic information about yourself or look for it in your medical record.	2	8		
206	This could hurt your chances to get or keep these types of insurance.	2	8		

Line Num	Sentence	# marked as Reassuring	# marked as Concerning	Regulatory Requirement	Req. met by Line Nums
207	**WHAT ARE MY OPTIONS?**	0	0		
208	Taking part in the Million American Study is your choice.	7	0	§ 46.116(b)(8)<br>§ 46.116(b)(4)<br>§ 164.508(c)(2) (ii)(a)	2; 208-214<br>208-209<br>208-214
209	You can choose to join or not.	6	2		
210	If you decide to join, you can change your mind at any time.	9	0	§ 46.116(c)(4)<br>§ 164.508(c)(2)(i)	210-214<br>210-213
211	Just let us know.	3	0		
212	If we have given out some of your materials for study, we cannot get them back.	3	0		
213	But we will send you a form so you can tell us what to do with your information and any of your sample that are left.	4	0		
214	If you decide not to participate, now or in the future, it will not affect your medical care.	4	0		
215	**WHO CAN ANSWER MY QUESTIONS?**	4	0	§ 46.116(d)(7)<br>§ 46.116(b)(7)	215<br>215

Please note: You may notice a few results above that perhaps seem incongruous, possibly even suggesting a misunderstanding. Qualitative data, however, indicate a reasonable basis for these responses. For example:

o Those who found project staff access to identifiers concerning (i) didn’t like that anyone would be able to see identifiers (i.e., they wanted their data to be totally anonymized, with no code to allow for re-identification), and/or (ii) thought the idea of a pledge was silly – that anyone could pledge to not do something but then do it anyway.

o As with the sentence about having blood drawn, those who found the sentence about risk of unintended access reassuring spoke about transparency. They were reassured that researchers were being open and honest about the risk, which made the endeavor seem more trustworthy in general.

S3. FIGURES

Figure 1. Number of sentences highlighted per participant (n = 32)

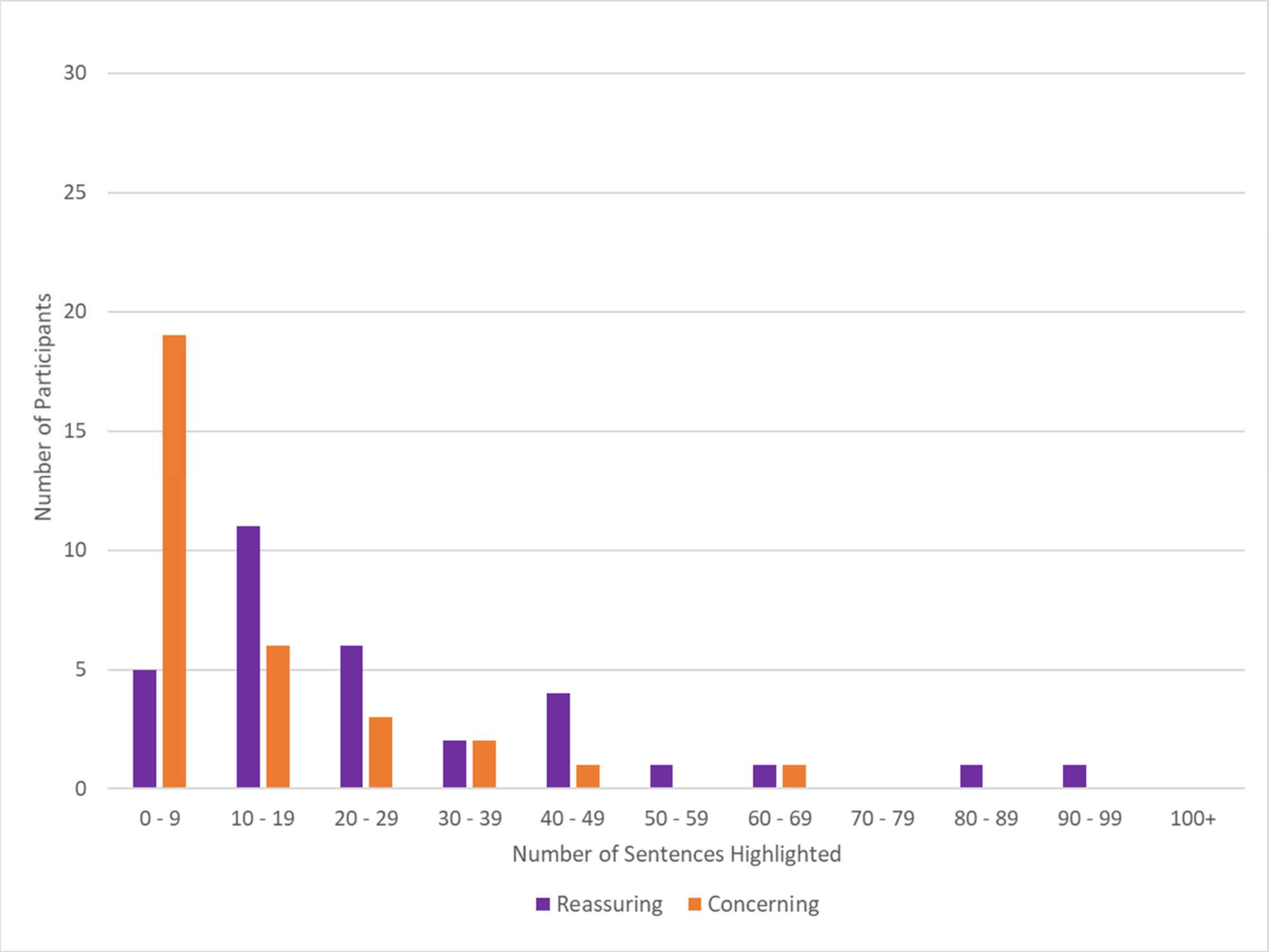

Figures 2a-b. Change in willingness to participate on 6-point scale (n = 32)

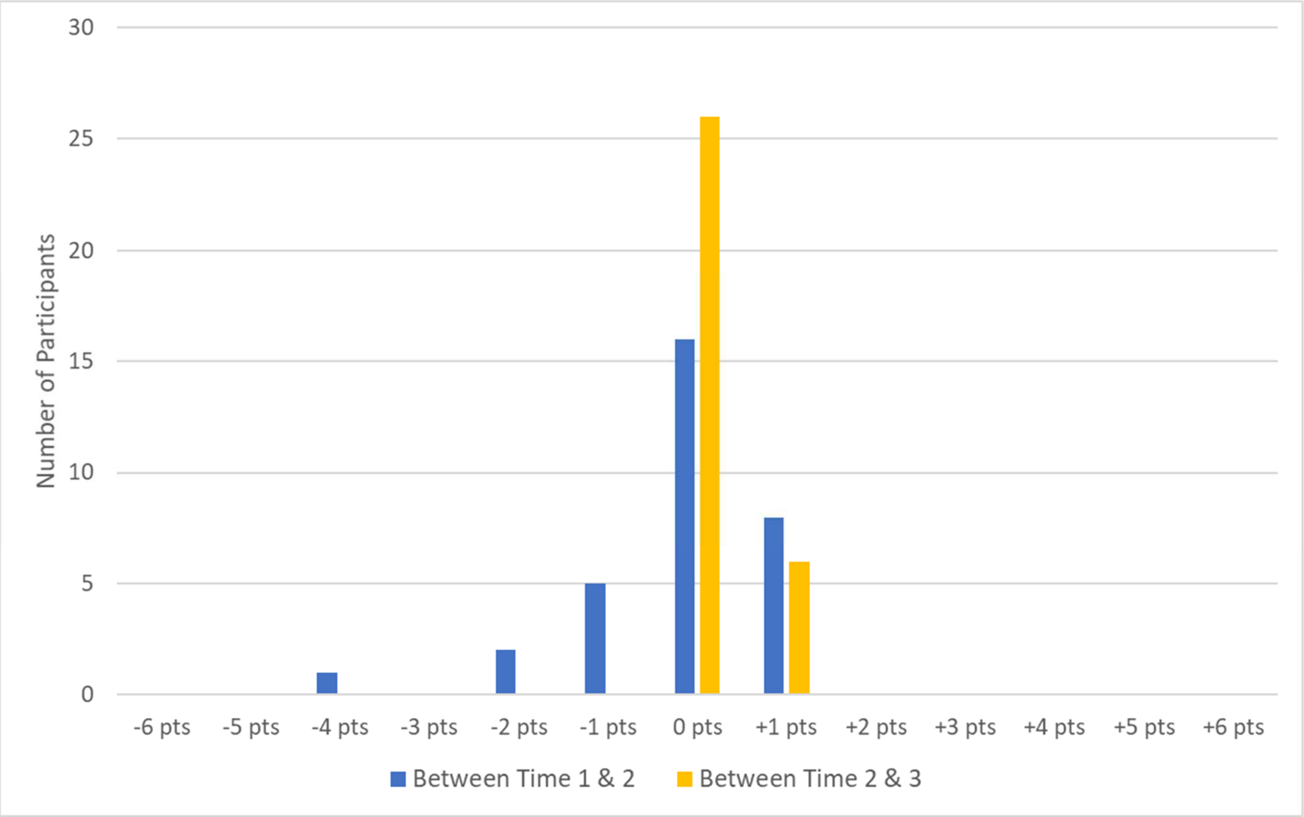

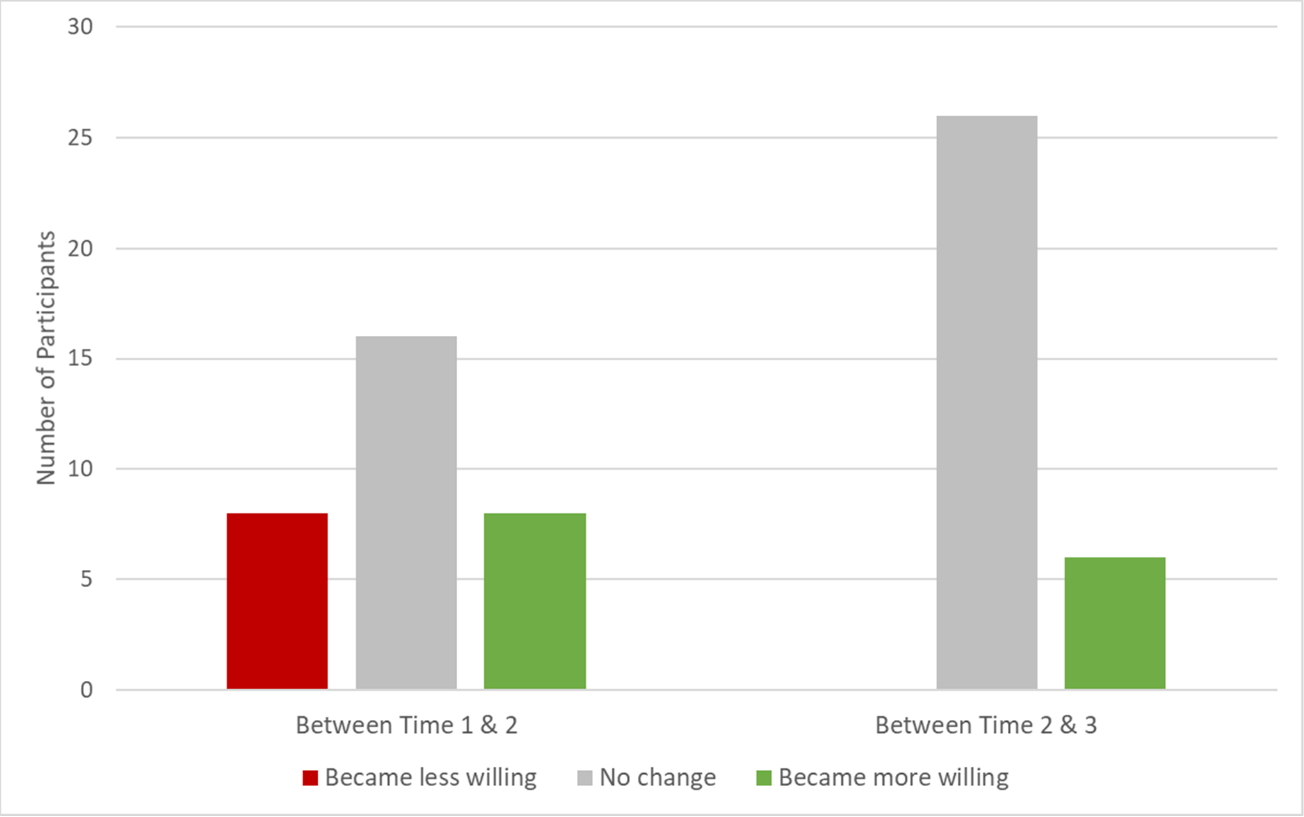

Time 1 = Initial review of consent form; **Time 2** = After highlighting exercise; **Time 3** = After values questions

Figure 3. Relationship between opinions on values questions and willingness to participate (n = 32)

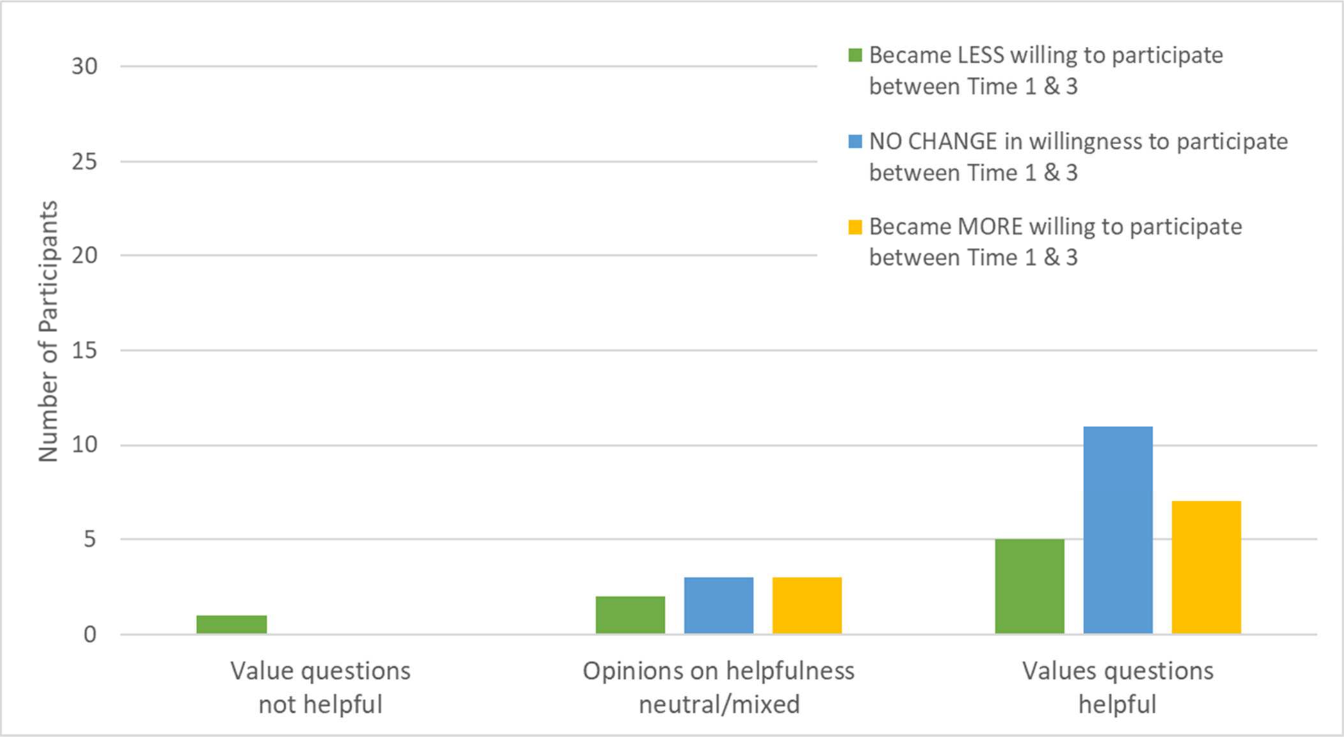

Supplement: Supplementary file 1 — Additional file 1. S1: Complete consent form with readability characteristics. S2: Complete consent form with highlight counts and regulatory justification. S3: Figures 1, 2a-b, and 3. [file 12874_2020_1001_MOESM1_ESM.pdf]
